# AI in Lung Nodules on Chest CT: Retrospective, Real-World Assessment of AI in Clinical Practice

**DOI:** 10.2196/86416

**Published:** 2026-05-28

**Authors:** Heyel Sodhi-Kalra, Shivangi Jha, Subba Digumarthy

**Affiliations:** 1 MGH Imaging Division of Thoracic Imaging, Department of Radiology, Massachusetts General Hospital, 55 Fruit Street, Boston, MA, 02114, United States, 1 6176434583

**Keywords:** lung nodules, artificial intelligence, computed tomography, CT, Lung Imaging Reporting and Data System, Lung-RADS, computer-aided detection

## Abstract

An FDA-cleared artificial intelligence tool demonstrated frequent discordance with radiologist reports for lung nodule assessment on chest CT.

## Introduction

While there are more than 1000 artificial intelligence (AI) tools with US Food and Drug Administration (FDA) clearance for clinical use, only a few have clinical implementation to improve the quality and efficiency of health care services due to worries over performance and meaningful use [1]. Errors in detection, number, and classification of lung nodules can lead to overdiagnosis with unnecessary medical expenses or underdiagnosis with missed lung cancer. We hypothesized that there are substantial discrepancies between the AI outputs and radiologists’ reports while using an FDA-cleared and clinically implemented AI tool for lung nodules seen on chest computed tomography (CT) exams. We evaluated the real-world performance of a clinically implemented AI tool for detecting, localizing, and measuring lung nodules on chest CT examinations.

## Methods

### Ethical Considerations

The institutional review board of Mass General Brigham approved the study (IRB protocol number 2020P003950).

### Study Design

We performed a retrospective evaluation of 650 consecutive, noncontrast chest CT examinations (age ≥21 y) processed with an FDA-cleared and clinically implemented AI tool for detecting lung nodules. Since the tool is not approved for pediatric use, we excluded patients less than 21 years of age. Postcontrast CT and CT angiography studies were also excluded. The CT examinations were identified from a review of our institutional radiology reports database and search engine (mPower, Microsoft Nuance). The AI tool detects (number of nodules), localizes (lung lobe location and image numbers), and measures (mean diameter and volume) lung nodules on chest CT exams. We recorded these AI outputs from the PACS (Picture Archiving and Communication System) into an Microsoft Excel file.

We then exported the corresponding radiology reports of all 650 CT examinations into an Excel file from our radiology reports database. All reports were generated by one of the 18 subspecialty thoracic radiologists with full access to the AI outputs and results at the time of their reporting. From the human-generated radiology reports, two study coinvestigators, (HSK, high school researcher; SJ, postdoctoral research fellow), extracted information regarding the number, location, attenuation category (solid, part-solid, and subsolid categories), and measurements of lung nodules.

Next, we compared the AI outputs with the recorded information from radiologists’ dictated radiology reports to determine exam-level concordance between AI and radiology reports for detection, average diameter measurement, lung lobe localization, and attenuation-based classification of lung nodules. A thoracic fellowship-trained subspecialty radiologist (SRD, 21 y of experience) adjudicated discrepant chest CT examinations with the corresponding AI image outputs and original report information to establish the reference standard and assess for the possibility of diagnostic error in the original radiology reports.

AI outputs were deemed discordant if they included false-positive or false-negative actionable nodules, wrong lobe localization of the dominant nodule, incorrect attenuation categorization, or incorrect measurement when compared with the adjudicated radiology report. Actionable nodules were defined as Lung-RADS-defining nodules (Lung Imaging Reporting and Data System), generally category 3 or higher (eg, solid nodules ≥6 mm or part-solid nodules ≥6 mm), whose detection, attenuation category, size, or location could affect clinical categorization or management. Nonactionable nodules were defined as non-Lung-RADS-defining nodules. Complete discordance referred to clinically meaningful misrepresentation of an actionable nodule or its defining features, including errors in attenuation, size, or location that could affect Lung-RADS categorization. Partial discordance referred to discrepancies involving non-actionable nodules or non-Lung-RADS-defining nodule features. AI outputs were deemed concordant when they were in complete agreement with the radiology report, following radiologist adjudication, for actionable and non-actionable nodules.

In addition, concordance and discordance rates were compared across sex and age categories using *χ*^2^ analysis, with *P*<.05. Confidence intervals for proportions were estimated with the Wilson score method.

## Results

Among the 650 chest CT examinations, agreement between the AI outputs and radiology reports for nodule presence, number, size, and location was seen in 104 cases (16.0%, 95% CI 13.4%‐19.0%). The remaining 546 per-exam showed at least one discrepancy between the AI outputs and the radiology reports (84.0%, 95% CI 81.0%‐86.6%). Of these discrepant per-exam, 340 involved actionable nodules and were categorized as complete per-exam discordance (52.3%, 95% CI 48.5%‐56.1%). Another 206 per-exam involved only non-actionable nodules or non-Lung-RADS-defining findings and were therefore categorized as per-exam partial discordance (31.7%, 95% CI 28.2%‐35.4%) (Table 1). After review of the discrepant cases, the adjudicating thoracic radiologist agreed with the original report interpretation in all but two examinations.

**Figure 1.**
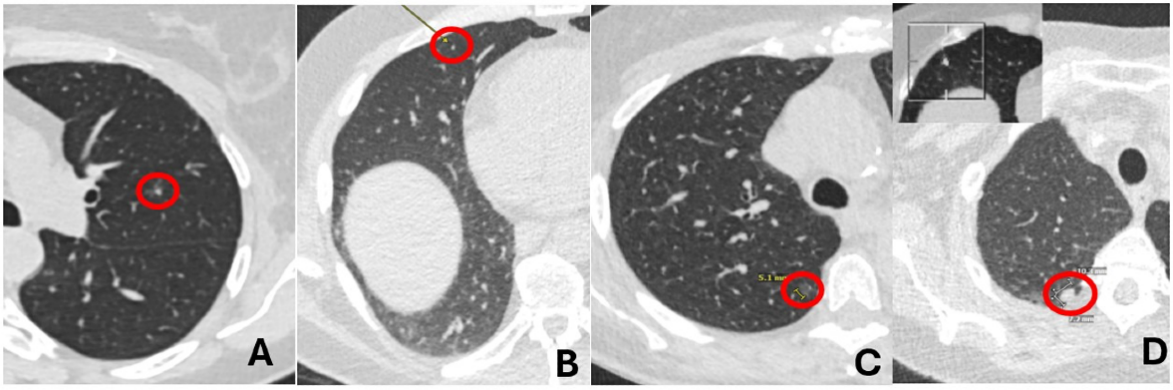
Transverse chest CT images demonstrating discrepancies between AI outputs and radiology reports for the following nodules: (A) A 4 mm left upper lobe nodule in a 50-year-old female patient was missed by the AI (complete discordance due to missed actionable nodule); (B) AI detected no nodules in a 57-year-old man with a 2 mm right middle lobe nodule in the radiology report (complete discordance due to missed actionable nodule); (C) AI measured the right upper lobe ground-glass nodule as 8 mm versus 5 mm in the radiology report (partial discordance: overestimation of non-actionable nodule); (D) AI reported an 8 mm right middle lobe nodule (inset) in a 71-year-old female patient, while the report described a 9 mm right upper lobe nodule as the actionable nodule (complete discordance from misdetection of actionable nodule). AI: artificial intelligence; CT: computed tomography.

Concordance and discordance categories did not differ significantly between female and male patients (*P*=.60) or between patients younger than 60 years and those aged 60 years or older (*P*=.79). Within the partially discordant group, disagreement involving both nodule size and location was the most frequent pattern (70/206, 34.0%), followed by disagreement involving nodule size and number (55/206, 26.7%). Examples of common discordance between AI and reports are presented in Figure 1.

**Table 1.** Distribution of concordant and discordant chest CT (computed tomography) examinations between AI outputs and radiologists’ reports. Percentages are presented with 95% CIs (Wilson method). Subcategories of complete and partial discordance are expressed as proportions of their respective groups.

Category	Number of CT exams	Percentage (95% CI)
Perfect concordance	104/650	16.0% (13.4‐19.0%)
Any discordance	546/650	84.0% (81.0‐86.6%)
Complete discordance (actionable nodules)	340/650	52.3% (48.5‐56.1%)
Nodule detection	180/340	52.9% (47.6‐58.2%)
Attenuation classification	40/340	11.8% (8.8‐15.6%)
Size	70/340	20.6% (16.6‐25.2%)
Lobe location	50/340	14.7% (11.3‐18.9%)
Partial discordance (non-actionable nodules)	206/650	31.7% (28.2‐35.4%)
Size+ Location	70/206	34.0% (27.9‐40.7%)
Size+ Number	55/206	26.7% (21.1‐33.1%)
Size Only	41/206	19.9% (15.0‐25.9%)
Number Only	14/206	6.8% (4.1‐11.1%)
Location + Number	12/206	5.8% (3.4‐9.9%)
Location Only	14/206	6.8% (4.1‐11.1%)

## Discussion

Most AI outputs for evaluating lung nodules were either completely (52.3%) or partially (31.7%) discordant with independent radiologist-adjudicated radiology reporting of lung nodules, emphasizing the suboptimal performance of AI in this real-world clinical setting. Our work cautions radiologists on the pitfalls of AI and the need for verification when using AI specifically for actionable nodule detection, attenuation categorization, and size measurement. FDA clearance does not guarantee AI performance or generalizability, a finding reported in other publications and attributed to several factors, including lack of transparency, robust multicenter testing, and comprehensive access to FDA clearance documents. Although most studies report the positive impact of AI on lung nodule detection [2,3], others have reported lower AI sensitivity for larger nodules (missed nodule size: AI, 11.5 mm vs radiologists, 4.7 mm [4]), lower specificity (AI, 77.5%‐87% vs radiologists, 87%‐91.7% [2]), and lower detection [5], size measurement [6,7], and accuracy for detecting subsolid nodules (area under the curve, 0.60‐0.72 [8]).

The main limitations of our study include lack of a power analysis, reliance on radiology reports with adjudication as the reference standard, use of an exam-level concordance framework rather than a prespecified diagnostic 2 × 2 framework for sensitivity and specificity, and inclusion of only a single vendor AI tool. An additional limitation is incorporation bias, as reporting radiologists had access to AI outputs during clinical interpretation. This could increase apparent agreement between AI outputs and radiology reports and therefore potentially underestimate true AI discordance. However, based on the observed low agreement between AI outputs and radiology reports, many reporting radiologists likely did not rely on AI outputs for describing lung nodule characteristics or presence.

In conclusion, radiologists must use caution when using an AI tool for evaluating lung nodules on chest CT due to its high rates of discordance with radiology reports.

## Abbreviations

CT: computed tomography
FDA: Food and Drug Administration
Lung-RADS: Lung Imaging Reporting and Data System
PACS: Picture Archiving and Communication System
